# Quantum‐Chemical Study of the FeNCN Conversion‐Reaction Mechanism in Lithium‐ and Sodium‐Ion Batteries

**DOI:** 10.1002/anie.201914760

**Published:** 2020-01-22

**Authors:** Kaixuan Chen, Marcus Fehse, Angelica Laurita, Jeethu Jiju Arayamparambil, Moulay Tahar Sougrati, Lorenzo Stievano, Richard Dronskowski

**Affiliations:** ^1^ Chair of Solid-State and Quantum Chemistry Institute of Inorganic Chemistry RWTH Aachen University 52056 Aachen Germany; ^2^ Institut Charles Gerhardt Montpellier CNRS Université de Montpellier 34095 Montpellier France; ^3^ Alistore—European Research Institute CNRS 80039 Amiens France; ^4^ Dutch-Belgian (DUBBLE) ESRF-The European Synchrotron 38043 Grenoble France; ^5^ Reseau sur le Stockage Electrochimique de l'Energie (RS2E) CNRS 80039 Amiens France; ^6^ Hoffmann Institute of Advanced Materials Shenzhen Polytechnic 7098 Liuxian Blvd Nanshan District Shenzhen China

**Keywords:** batteries, carbodiimides, computational chemistry, density functional calculations, lithium ions

## Abstract

We report a computational study on 3d transition‐metal (Cr, Mn, Fe, and Co) carbodiimides in Li‐ and Na‐ion batteries. The obtained cell voltages semi‐quantitatively fit the experiments, highlighting the practicality of PBE+U as an approach for modeling the conversion‐reaction mechanism of the FeNCN archetype with lithium and sodium. Also, the calculated voltage profiles agree satisfactorily with experiment both for full (Li‐ion battery) and partial (Na‐ion battery) discharge, even though experimental atomistic knowledge is missing up to now. Moreover, we rationalize the structural preference of intermediate ternaries and their characteristic lowering in the voltage profile using chemical‐bonding and Mulliken‐charge analysis. The formation of such ternary intermediates for the lithiation of FeNCN and the contribution of at least one ternary intermediate is also confirmed experimentally. This theoretical approach, aided by experimental findings, supports the atomistic exploration of electrode materials governed by conversion reactions.

## Graphical abstract

**A clear case**: A computational study on the conversion‐reaction mechanism of FeNCN in Li‐ and Na‐ion batteries yields calculated voltage profiles which agree well with experiments, and their characteristic behavior is well explained by chemical‐bonding and Mulliken‐charge analysis. Furthermore, a predicted ternary intermediate with composition Li_2_Fe_2_(NCN)_3_ during lithiation is confirmed by operando XAS analysis.

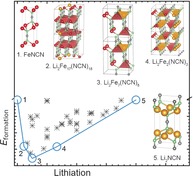

## Introduction

With the fast advancement in science and technology, and even faster growing demands by society, the field of energy production and storage is becoming critically important. Regarding high‐density energy‐generating approaches, there has been continuous progress in advanced nuclear power such as breeder/burner technology, thorium reactors, generation IV, and even small modular fission concepts.^1^ With respect to low‐density energy production and storage, extensive attention has been directed at research concerning solar cells,^2^ supercapacitors,^3^ thermoelectrics,^4^ Li‐ and Na‐ion batteries (LIBs/NIBs),^5^ as well as related fields. For reasons of convenience and the perception of the general public,^6^ LIBs are widely used in various portable devices and electric vehicles,^7^ but this only works as long as cheap primary electric energy is produced to feed them. Technically speaking, the performance of a Li/Na‐ion battery, determined, for example, by its cell voltage, capacity, and reversible cycling number, essentially depends on the assembly of the electrodes and electrolytes.^8^

When viewing the frontier research of these electrodes from a somewhat wider perspective, two kinds of mechanisms seem to dominate, namely intercalation and the conversion reaction.5b Typical commercial electrodes such as LiCoO_2_ and graphite follow the intercalation mechanism in which Li ions intercalate into or de‐intercalate from the electrode host without significantly altering its original structure. Hence, the battery capacity is essentially restricted by the available vacancies in an electrode host to accommodate foreign Li ions. In the conversion‐reaction mechanism, however, Li ions occupy not only the vacancies but also the sites of the metal cations, accompanied by a deposition of elemental metal particles. Because of this, drastic structural transformations may occur upon charge/discharge cycling, even leading to the forming of new phases. Compounds such as transition‐metal oxides, nitrides, sulfides, and fluorides are generally governed by a conversion reaction.5b, 9

In recent years, the family of transition‐metal carbodiimides (TMC)5a, 10 has attracted increasing interest. The TMC family adopts the generic formula M_*x*_(NCN)_*y*_, where M denotes a transition‐metal cation and the NCN^2−^ carbodiimide is the “divalent nitride” anion, mimicking the O^2−^ anion by a nitrogen functionality. Its members, including Cr_2_(NCN)_3_,^11^ MnNCN,^12^ FeNCN,^13^ CoNCN,^14^ and NiNCN,^15^ have been successfully fabricated by “soft chemistry” (metathesis) routes and furthermore, they turned out to be excellent anode candidates possessing a high reversible capacity upon charge/discharge cycling in Li‐ and Na‐ion batteries.^5^, ^16^ Nevertheless, the detailed reaction pathway and unsolved structural transformations that occur upon charge/discharge cycling are difficult to detect experimentally because of the often amorphous nature of these materials. Hence, the main focus of this contribution is to carry out an in‐depth density functional theory (DFT) study of the conversion‐reaction mechanism of typical FeNCN as a LIB (NIB) electrode material by employing advanced DFT calculations combined with operando X‐ray absorption spectroscopy (XAS).

## Results and Discussion

We calculated the average cell voltages^17^ of the TMC (TM=Cr, Mn, Fe, Co) vs. pure Li and Na metals using the PBE and PBE+*U* functionals (see the Supporting Information).^18^ Pure PBE produces less reliable voltage data (the largest error being 1.2 V for CoNCN in a LIB) because the *U* correction is necessary to model the highly correlated TM electrons.^19^ As shown in Table S2 (Supporting Information), introducing the *U* correction^20^ effectively decreases the voltage errors (to about 0.8 V for CoNCN in a LIB), so we chose PBE+*U* for a large‐scale study.

**Figure 1:**
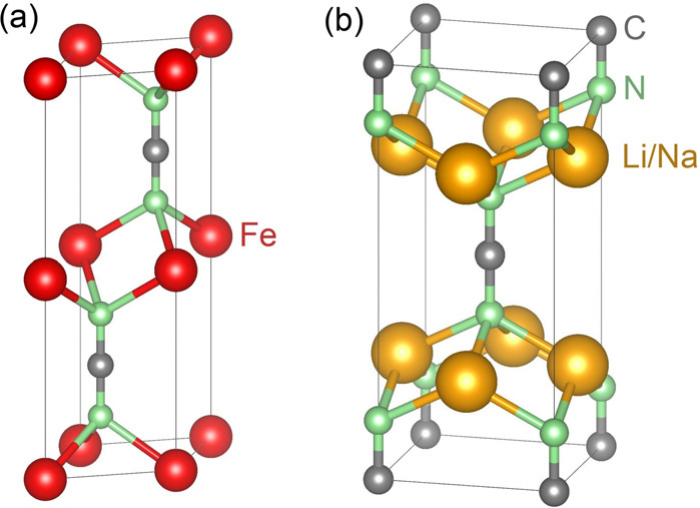
Crystal structures of a) FeNCN and b) Li_2_NCN/Na_2_NCN.

Since we focus on the reaction pathways and voltage profiles during the charge/discharge process for the FeNCN archetype in Li/Na‐ion batteries, the crystal structures of the fully delithiated (desodiated) compound FeNCN and the lithiated (sodiated) compound Li_2_NCN (Na_2_NCN) should be analyzed first (Figure 1). FeNCN crystallizes in the hexagonal system with *P*6_3_/*mmc* symmetry, that is, octahedral coordination for Fe and trigonal‐prismatic coordination for NCN, resembling the nickel arsenide motif. Li_2_NCN (Na_2_NCN), however, adopts a tetragonal *I*4/*mmm* structure with tetrahedral coordination for Li (Na) and a quadratic‐prismatic coordination for NCN. Like the related oxide compounds, transition‐metal carbodiimides follow a conversion‐reaction mechanism when used as electrode materials. That means that drastic structural transformations occur during the charge/discharge process. It is unclear up to which degree the crystal structures of the intermediate compounds may resemble FeNCN or Li_2_NCN in a LIB. It is therefore far from trivial to correctly deduce the atomistic structural details of these ternaries denoted as Li–Fe–NCN, in particular because reliable experimental models (for example, from X‐ray diffraction) are missing. Because of this, we tentatively used four kinds of structural bases for a thorough survey of possible Li–Fe–NCN ternaries: two from divalent carbodiimides,^21^ namely the NiAs‐type FeNCN and the NaCl‐type MnNCN, one from Li_2_NCN, and one from Li‐containing metal oxides such as CuLi_2_O_2_ (where one needs to replace O^2−^ anions with NCN^2−^ groups).

During the discharge process in a Li‐ion half‐battery with Li metal as the anode, the cathode (FeNCN) accommodates both Li^+^ cations (through the electrolyte) and electrons (through an external loop) to form Li–Fe–NCN ternaries. Note that an oxidation reaction in the positive FeNCN electrode forming Fe^3+^ cations is prevented during lithiation (cathodic conditions), which rather leads to the reduction of the material producing the deposition of elemental Fe^0^. To maintain electrical neutrality in a Li–Fe–NCN ternary, Fe^2+^/Fe^0^ reduction and Li^0^/Li^+^ oxidation must proceed in a 1:2 proportion since one Fe^2+^ acts as two Li^+^ in the charge compensation. In other words, a cathode host accommodates two Li and precipitates one Fe simultaneously. That is, the intermediate compounds, whatever they may be, share the generic chemical formula Li_*x*_Fe_(1−*x*/2)_NCN with 0<*x*<2. We enforced this confinement due to charge balance when establishing the model‐type intermediate compounds^22^ as derived from the aforementioned four structural bases and as tabulated in Table 1.

**Table 1:** Total number of computations to survey the possible Li–Fe–NCN (Na–Fe–NCN) ternary compounds.

structural basis	space group	no. of calculated compounds	
		*Li‐ion*	*Na‐ion*
FeNCN	*P*6_3_ */mmc* (no. 194)	17	17
Li_2_NCN/Na_2_NCN	*I*4*/mmm* (no. 139)	23	16
MnNCN	*R* $\bar{3}$ *m* (no. 166)	10	10
CuLi_2_O_2_	*Immm* (no. 71)	11	11

After full optimizations, the structures were used to perform electronic self‐consistency calculations. To examine the stability of each intermediate compound, its formation energy *E*_formation_ was calculated, which is defined as the energetic difference between an intermediate compound Li_*x*_Fe_(1−*x*/2)_NCN and its corresponding linear combination of Li_2_NCN (fully lithiated) and FeNCN (fully delithiated):

(1) $$E_{formation}=E\left({Li}_{x}{Fe}_{\left(1-x/2\right)}NCN\right)-\frac{x}{2}E\left({Li}_{2}NCN\right)-\left(1-\frac{x}{2}\right)E\left(FeNCN\right)$$

**Figure 2:**
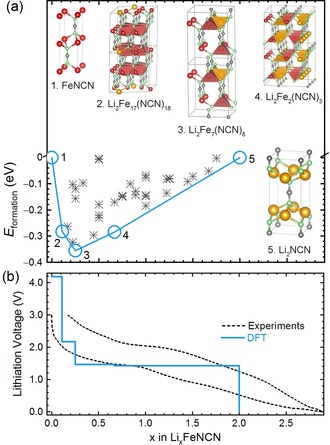
a) Formation energies (crosses) of the calculated Li–Fe–NCN ternary compounds together with the energy convex hull as a blue line and b) voltage profile of FeNCN upon lithiation in a LIB.

Figure 2 a shows the formation energies of all calculated Li_*x*_Fe_(1−*x*/2)_NCN compounds at various configurations. By convention, the more negative the formation energies, the more stable the compounds. All unstable (or metastable) compounds with positive formation energies were not included in Figure 2 a. For convenience, a line connecting the compounds that are most stable at each configuration is also depicted in blue (minimum‐energy curve or energy convex hull).^23^ Given full thermodynamic control, the reaction pathway during the charge/discharge process would have to follow this line (related phase diagram shown in Figure S3).

Each voltage pair (given by two intermediate compounds with adjacent Li content in the phase diagram) gives a voltage plateau, and a voltage profile can be obtained by combining all voltage plateaus. For example, given a voltage pair of two intermediate compounds written as ${Li}_{x_{1}}{Fe}_{\left(1-x_{1}/2\right)}NCN$ and ${Li}_{x_{2}}{Fe}_{\left(1-x_{2}/2\right)}NCN$ (taking *x*_1_<*x*_2_ with FeNCN as a cathode host in a LIB), their reaction reads:

(2) $$\left(x_{2}-x_{1}\right)Li+{Li}_{x_{1}}{Fe}_{\left(1-x_{1}/2\right)}NCN\;\to {Li}_{x_{2}}{Fe}_{\left(1-x_{2}/2\right)}NCN+\frac{1}{2}\left(x_{2}-x_{1}\right)Fe,$$

so that the voltage plateau *Φ*(*x*_1_, *x*_2_) results, similar to that from M_x_(NCN)_*y*_ to Li_2_NCN, in:

(3) $$\Phi \left(x_{1},x_{2}\right)=\;-\frac{\Delta G}{\left(x_{2}-x_{1}\right)zF}\approx -[E\left({Li}_{x_{2}}{Fe}_{\left(1-x_{2}/2\right)}NCN\right)+\frac{1}{2}\left(x_{2}-x_{1}\right)E\left(Fe\right)-E\left({Li}_{x_{1}}{Fe}_{\left(1-x_{1}/2\right)}NCN\right)-\left(x_{2}-x_{1}\right)E\left(Li\right){]\left[\left(x_{2}-x_{1}\right)zF\right]}^{-1}$$

Figure 2 b depicts the theoretical voltage profile for the archetype FeNCN using a blue solid line, corresponding to the minimum‐energy curve in Figure 2 a. The staircase shape originates from the limited number of stable compounds obtained from the also limited number of DFT calculations.

For conversion‐reaction mechanism materials, there is clearly a large experimental difference between the first and second lithiation, but the subsequent cycles are identical.^24^ Indeed, during the first lithiation process, large and well‐crystallized particles are transformed into nanosized and amorphous particles. Only after this irreversible morphological change, the lithiated material becomes the true cycling material. Thus, the experimental profile of FeNCN at the second cycle at a rate of C/50 (corresponding to the reaction of one mole of Li per mole of FeNCN in 50 h) is depicted (as a dashed line) for comparison.

In the theoretical data, an abnormally high voltage plateau (about 4.2 V vs. Li^+^/Li^0^) is observed at a low Li content (*x*<0.2). This effect is a computational artifact since an extremely large supercell would be required to perfectly distribute Li sites and Fe vacancies at very low Li content, which cannot be modeled with our limited computational resources. Fortunately, the requirement is much weaker for a medium Li content, which makes the voltage profile more reliable in that range. Another divergence is the maximally available Li content *x*, which should be 2 (theoretically) when the fully lithiated compound Li_2_NCN forms. In the experiment, *x* can become larger than 2, and actually goes up to about 2.8 because of side reactions. We note that the electrode is made not only of FeNCN but also includes 40 w % of carbon and binder (CMC, carboxymethyl cellulose). Therefore, the reasons for the larger Li content may include the formation of a passivation layer (SEI, solid–electrolyte interface) on the active material and electrolyte degradation. This is also the reason why the capacity of the first lithiation is always higher than that of subsequent cycles. For FeNCN, the SEI (or electrolyte degradation) seems to be largely reversible, and seemingly keeps cycling at more than 2 Li. This phenomenon is also observed in transition‐metal oxides.^14^ Despite the two unavoidable divergences, the calculated voltage profile agrees well with experiment and nicely reproduces a similar voltage plateau for a wide Li range (0.2<*x*<2.0).

**Figure 3:**
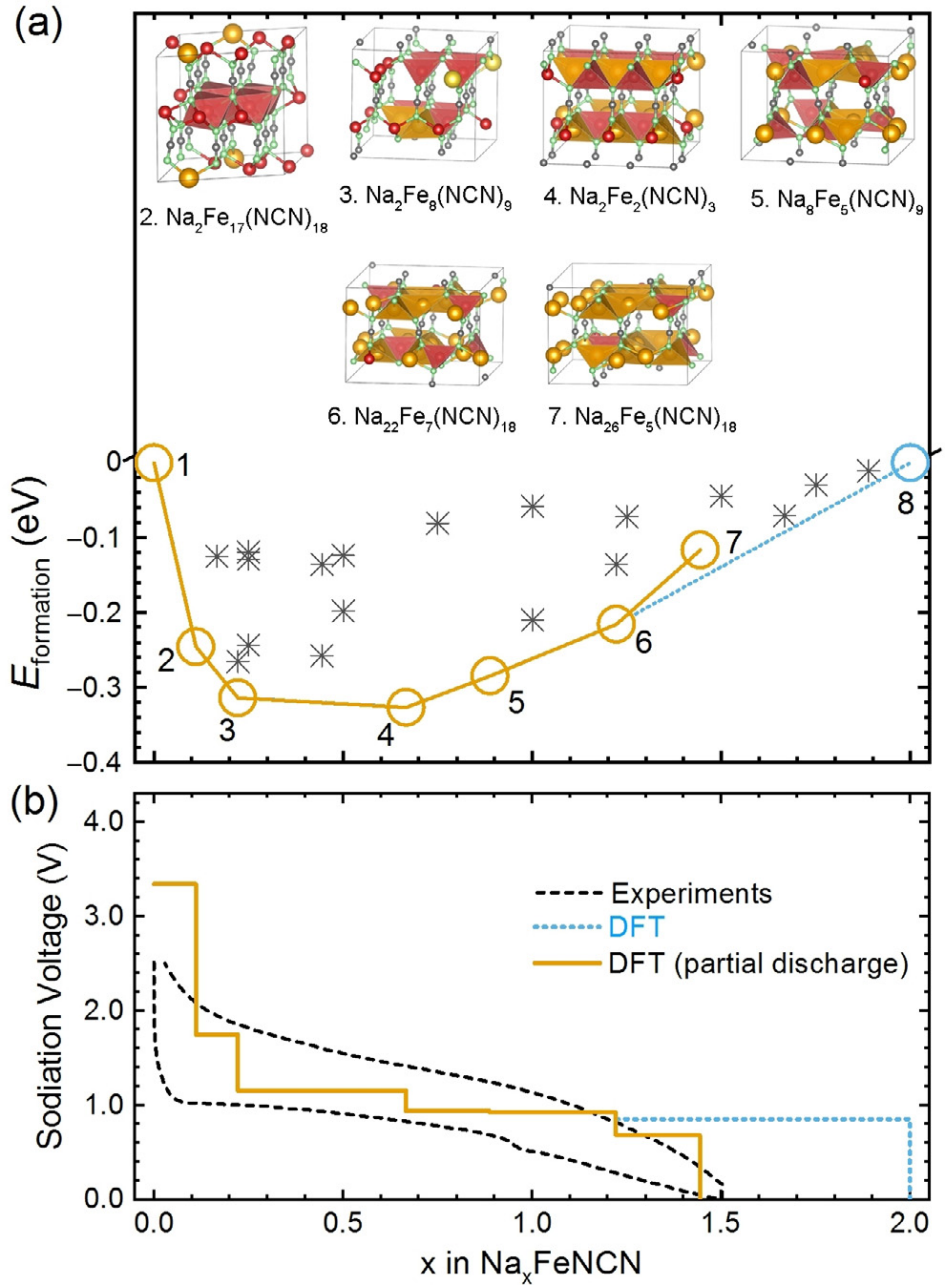
a) Formation energies (crosses) of the calculated Na–Fe–NCN ternary compounds together with the energy convex hull as a yellow line and b) voltage profile of FeNCN upon lithiation in a NIB.

Figure 3 a,b depicts the minimum‐energy curve and voltage profile for the FeNCN archetype in a NIB, obtained with the same computational approach as used for the LIB before. An analogous high voltage plateau (about 3.4 V vs. Na^+^/Na^0^) appears at a low Na content (*x*<0.2), similar to the LIB. The maximum experimentally achievable Na content, however, is only *x*=1.5. This is because Na fails to react with all FeNCN in experiments, probably due to kinetic hindrance, so there is only a partial reaction (or partial discharge). The SEI (or electrolyte degradation) effect is the same as in a LIB, but with less reversible capacity to form the active material. If we set *x*=1.5 as the theoretical maximum Na content, that is, the discharge process ends at Na_6_Fe(NCN)_4_ and not Na_2_NCN, we end up with a new voltage profile for a partial discharge. It is reassuring that this new profile, depicted as a solid orange line in Figure 3 b, agrees nicely with the experimental result.

**Figure 4:**
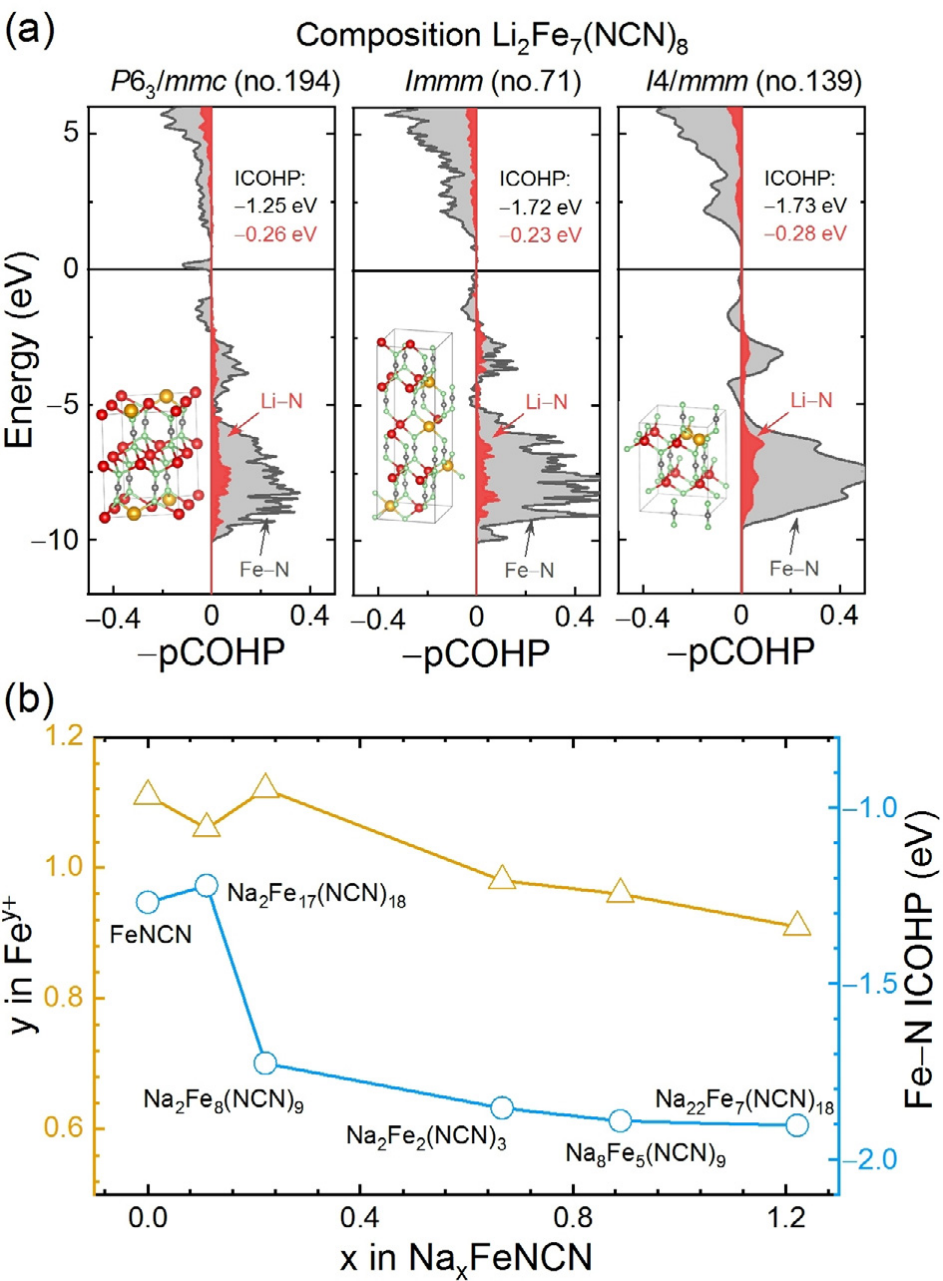
a) Chemical‐bonding analysis of the Fe−N and Li−N bonds in three Li_2_Fe_7_(NCN)_8_ structural models. The Fermi level is set to zero. b) Mulliken atomic charges of Fe and ICOHP data of the Fe−N bonds in the energetically favorable Na–Fe–NCN ternaries.

For further interpretation and understanding of the aforementioned findings, chemical‐bonding analysis was employed. In the cathode of a LIB or NIB, the ideal reaction pathway (or minimum‐energy curve) starts at FeNCN, proceeds through a variety of intermediate (amorphous) compounds, and ends at Li_2_NCN or Na_2_NCN. For an intermediate compound with a certain stoichiometric configuration, for example, Li_2_Fe_7_(NCN)_8_, different crystal‐structure models were computationally established. Taking a thermodynamic point of view, the one crystallizing with the lowest formation energy was preferred but we note that other metastable variants with higher formation energies but lower activation barriers are also possible according to Ostwald's step rule.^25^ In the following, we rationalize the occurrence of structural polymorphs from the aspect of chemical bonding. Figure 4 a depicts the composition Li_2_Fe_7_(NCN)_8_ (corresponding to a Li content of *x*=0.25) for which three structural models (*P*6_3_/*mmc*, *Immm*, and *I*4/*mmm*) were established. Among these, *I*4/*mmm* has the lowest formation energy. Previous research indicates that the structural preference in carbodiimides can be determined from chemical‐bonding analysis.^21^, ^26^ Figure 4 a presents a crystal orbital Hamilton population (COHP) analysis of both Fe−N and Li−N bonds in the three Li_2_Fe_7_(NCN)_8_ models adopting *P*6_3_/*mmc*, *Immm*, and *I*4/*mmm* symmetry. Negative (or positive) COHP values indicate energy‐lowering bonding (or energy‐increasing anti‐bonding) interactions. It is apparent that bonding interactions dominate in the low‐energy valence‐band region whereas anti‐bonding states appear in the unoccupied conduction band, but also just below the Fermi level. For a quantitative assessment, all COHP values were integrated up to the Fermi level to yield ICOHP data for the Fe−N and Li−N bonds and averaged over all corresponding bonds in the unit cell. It turns out that the *I*4/*mmm* model of Li_2_Fe_7_(NCN)_8_ not only holds the strongest bonding interactions for both Fe−N and Li−N bonds but also has the lowest formation energy. Hence, the chemical‐bonding analysis is useful to deduce where the structural preference of the intermediate compounds originates from.

As discussed above, the cell voltage for FeNCN originates from the potential difference between the Fe^2+^/Fe^0^ and Li^+^/Li^0^ (Na^+^/Na^0^) redox couples in a LIB (NIB). An ideal voltage profile displays a flat plateau with a theoretical voltage of 1.6 V (1.1 V) in a LIB (NIB), as tabulated in Table S2. The actual profile, however, exhibits a monotonous decrease in voltage upon lithiation (sodiation) both experimentally and theoretically. We rationalize this decreasing character from a monotonous decrease in the atomic charge of Fe, expressed as *y* in Fe^*y*+^ in the energetically low‐lying intermediate ternaries, and evaluated from Mulliken charge analysis in Figure 4 b. To zeroth order, the higher the cationic charge, the stronger the reduction power. Indeed, the Mulliken charge of Fe decreases from 1.1 in FeNCN to 0.9 in Na_22_Fe_7_(NCN)_18_, seemingly leading to a decrease in the Fe^*y*+^/Fe^0^ redox potential; note that these (basis‐set‐independent) Mulliken charges were numerically projected from the entire wavefunction expanded from plane waves.^27^ Figure 4 b also demonstrates a more negative ICOHP progression for the Fe−N bonds, but now upon sodiation in a NIB. This indicates an almost continuous increase in covalency for the Fe−N bonds in the energetically low‐lying intermediate ternaries, another confirmation of a smaller amount of electron density transferred from Fe to N and, thus, a decrease in the charge of Fe.

**Figure 5:**
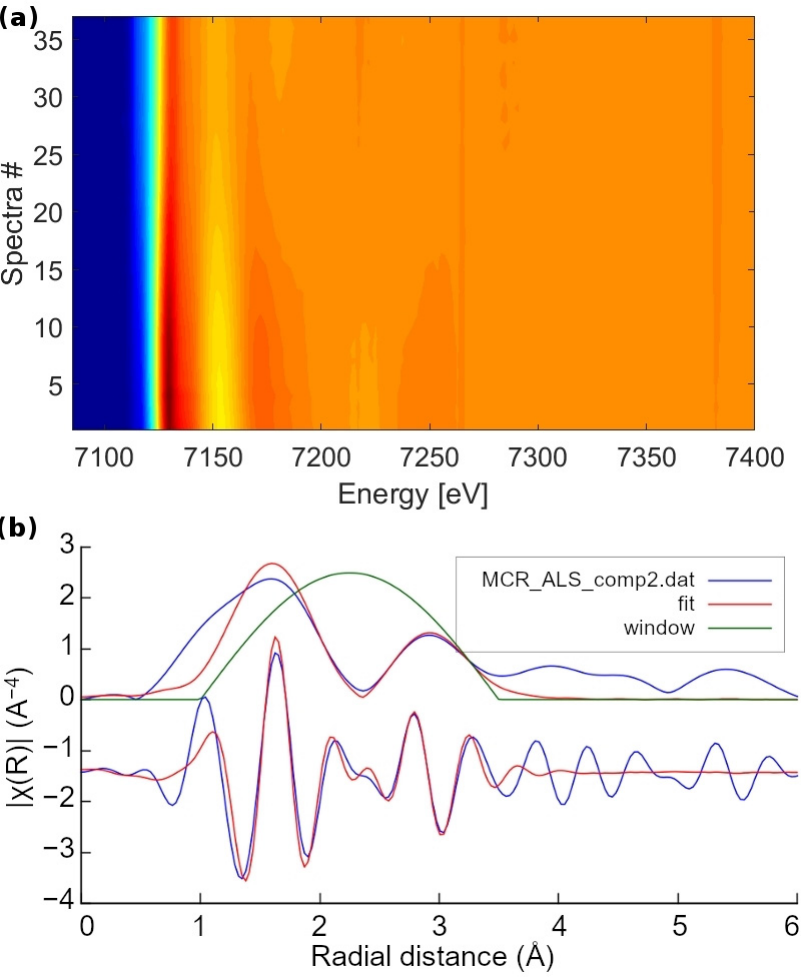
a) Contour plot of the entire Fe K‐edge XAS operando data set revealing the evolution of the XANES and EXAFS of the Fe K‐edge upon electrochemical discharge vs. Li; b) Fourier transform of the EXAFS of the Fe K‐edge of intermediate component 2 obtained via MCR‐ALS and EXAFS fit with a fitting window.

At last, the lithiation of FeNCN was followed experimentally by operando Fe K‐edge XAS in order to corroborate or falsify the theoretical findings.^28^ In particular, the extended fine structure of the XAS spectrum (EXAFS)^29^ is very sensitive to the modification of the nearest‐neighbor shell of the Fe atoms and is hence used to validate the DFT‐predicted ternary intermediates. The complete operando data set depicting the evolution of Fe K‐edge XAS spectra acquired during the first electrochemical discharge of FeNCN vs. Li is presented as a contour plot in Figure 5 a. The plot shows a decrease of the edge‐energy intensity accompanied by a shift to lower edge energy, reflecting the effective chemical reduction of the Fe atoms.

Principal component analysis (PCA)^30^ indicates that three independent principal components are needed to reproduce the entire data set, yielding 99.9 % total variance, as shown in Figure S4. Subsequently pure spectral components were reconstructed via multivariate curve resolution alternating least squares (MCR‐ALS).^31^ The EXAFS spectra indicate that component 1 and component 3 can be easily fitted with the structure of pristine FeNCN and the iron nanoparticles, as shown in Figure S6 and S7, respectively. Component 2 represents the ternary intermediate, which reaches its maximum concentration at about one third of the first discharge (Figure S5 b), and can therefore be successfully fitted by the theoretical structure of Li_2_Fe_2_(NCN)_3_, in line with the amount of reacted lithium. The compound Li_2_Fe_2_(NCN)_3_, structurally derived from Li_2_NCN, yields satisfactory results, as shown in Figure 5 b and Table 2. The quantum‐chemical bonding analysis in Figure S8 also indicates that the Fe−N bonds in the Li_2_Fe_2_(NCN)_3_ structure derived from the Li_2_NCN supercell possess a more negative ICOHP value (meaning stronger bonding, −1.84 eV) than the ones in the FeNCN supercell (−1.46 eV). In other words, the good agreement of the DFT‐predicted structure with the experimental spectra confirms the formation of an intermediate yet amorphous phase compatible with the local structure of Li_2_Fe_2_(NCN)_3_ during the conversion reaction of FeNCN vs. Li. Unravelling such transient phases in complex reaction mechanisms is a valuable asset of the MCR‐ALS approach, was recently shown for sodiation of SnSb.^32^

**Table 2:** EXAFS fitting parameters of MCR‐ALS component 2 of the Fe K‐Edge.

shell	coord. no.	theor. distance (Å)	exp. distance (Å)	*σ* ^2^
Fe−N	4	2.07	2.14(1)	0.007(2)
Fe−C	3	2.93	3.06(5)	0.01(1)
Fe−Fe	2	3.46	3.27(1)	0.007(3)

## Conclusion

In conclusion, given the new family of 3d transition‐metal carbodiimides which have experimentally turned out as excellent candidates for Li‐ and Na‐ion batteries, we have demonstrated the first computational attempt to model its electrochemical mechanism. A first‐principles study on the conversion‐reaction mechanism of the archetype FeNCN with Li and Na during charge/discharge has been performed by simulating a total of 61 (54) configurations of Li–Fe–NCN (Na–Fe–NCN) ternaries. Based on their structures, spin‐polarized PBE+*U* calculations gave access to voltage profiles which agree nicely with experiment for both full (LIB) and partial (NIB) discharge. The origins of the energetically preferred structures adopted in the intermediate compounds were rationalized by chemical‐bonding analysis. Additionally, the continuously lowering voltage profile was also mirrored in the COHP progression and the Mulliken charge transfer. Operando XAS analysis performed during the lithiation of FeNCN substantiates the results of these calculations, confirming the formation of an intermediate species with a structure compatible with the theoretically predicted transient phase Li_2_Fe_2_(NCN)_3_. Finally, this study offers a new challenge to chemists in the preparation of metastable Li–Fe–NCN intermediates.

## Conflict of interest

The authors declare no conflict of interest.

## Supporting information

As a service to our authors and readers, this journal provides supporting information supplied by the authors. Such materials are peer reviewed and may be re‐organized for online delivery, but are not copy‐edited or typeset. Technical support issues arising from supporting information (other than missing files) should be addressed to the authors.

Supplementary
