# Supplementary material for: Quantum‐Chemical Study of the FeNCN Conversion‐Reaction Mechanism in Lithium‐ and Sodium‐Ion Batteries
Source: Angew Chem Int Ed Engl. 2020 Jan 22;59(9):3718–23. doi: 10.1002/anie.201914760 (PMC7065120; doi:10.1002/anie.201914760)
Supplement: Supplementary file 1 — Supplementary [file ANIE-59-3718-s001.pdf]

Angewandte Chemie
Eine Zeitschrift der Gesellschaft Deutscher Chemiker

Supporting Information

Quantum-Chemical Study of the FeNCN Conversion-Reaction Mechanism in Lithium- and Sodium-Ion Batteries

Kaixuan Chen, Marcus Fehse, Angelica Laurita, Jeethu Jiju Arayamparambil, Moulay Tahar Sougrati, Lorenzo Stievano, and Richard Dronskowski**

anie_201914760_sm_miscellaneous_information.pdf

Contents

1. Experimental Section 2

2. Choice of PBE+*U* model 3

3. Calculated theoretical phase diagram 4

4. *Operando* XAS data analysis 5

5. Chemical bonding analysis of $Li_2Fe_2(NCN)_3$ models 7

6. References 8

1. Experimental Section

General computational methods. Spin-polarized first-principles calculation were performed using the Vienna ab-initio simulation package (VASP).[1] Projector-augmented-wave (PAW) potentials with the exchange-correlation functional provided by Perdew–Burke–Ernzerhof (PBE) were adopted. Γ-centered Monkhorst–Pack *k*-meshes were generated using the Vaspkit tool with a recommended value of 0.04 2π/Å, which produces a 9 × 9 × 3 (7 × 7 × 3) *k*-mesh for the unit cell of FeNCN (Li_2NCN). The plane-wave energy cutoff was set to a fairly high 600 eV within the DFT+*U* framework while the *U* values were obtained from a previous study on the 3*d* transition-metal oxides.[2] In numbers, we used 3.5 eV, 3.9 eV, 4.0 eV and 3.4 eV for Cr, Mn, Fe and Co, respectively. To model the possible intermediate compounds with various compositions, supercells with maximum sizes up to 3 × 3 × 1 were employed based on the crystal structures of FeNCN and Li_2NCN/Na_2NCN; for those deduced from Li_2CuO_2 and MnNCN, the supercells were sized as 2 × 2 × 1. A total of 61 (54) configurations of intermediate compounds during the charge/discharge process in a LIB (NIB) was established. The Ewald summation technique, as implemented in the Pymatgen tool[3], was used to search the optimized sites/holes distribution which gives the lowest electrostatic energy.

From these DFT calculations one may obtain, at least in principle, the theoretical cell voltage $\overline{\Phi}$ of a cathodic $M_x(\mathrm{NCN})_y$ vs an anodic Li/Na metal given the total electronic-energy difference Δ*E* as based on the following exemplary reaction equation:

$$M_x(\mathrm{NCN})_y + 2y\ \mathrm{Li} \xrightarrow{\mathrm{discharge}} x\ M + y\ \mathrm{Li_2NCN}$$

$$\overline{\Phi} = -\frac{\Delta G}{zF} \approx -\frac{\Delta E}{zF}$$

Here, *z* denotes the electron transfer that has been carried by each cation (in Li/Na-ion batteries, *z* = 1), and *F* is the Faraday constant (one mole of electrons).[4] The variation of the Gibbs free energy Δ*G* consists of three parts, the largest of which is the electronic-energy difference Δ*E*. In addition, there are the *P*Δ*V* volume and the *T*Δ*S* entropy terms which may safely be neglected, because of their small proportion (about 1%),[5] as long as we deal with low-temperature solid-solid reactions. Hence, for reasons of simplicity and the moment being we approximate $\Delta G \approx \Delta E$. The structures of Li, Na, and the 3*d* transition metals (Cr, Mn, Fe, Co) used in the formation-energy calculations are shown in Table S1. For self-consistency calculations, the energy convergence was set to 10^{-6} eV, that is, 0.0005 meV/atom.

Table S1: Crystal-structure parameters of metals used for formation-energy calculations.

metal	space group	*a* (Å)
Li	$Im\bar{3}m$ (no. 229)	3.44
Na	$Im\bar{3}m$ (no. 229)	4.19
Cr	$Im\bar{3}m$ (no. 229)	2.86
Mn	$Im\bar{3}m$ (no. 229)	3.37
Fe	$Im\bar{3}m$ (no. 229)	2.94
Co	$P6_3/mmc$ (no. 194)	2.46

To rationalize all following results in terms of chemistry, the chemical-bonding and charge-transfer analyses were performed with LOBSTER by means of the “pbevaspfit2015” contracted Slater-type basis set.[6]

Experimental methods. FeNCN active electrode material was prepared as described in detail elsewhere.[7] Self-supported electrode pellets with a composition of 60% active material, 20% CMC binder, 10% carbon black and 10% vapor grown carbon fibers were used. Fe K-edge XAS measurements were carried out in a specifically designed electrochemical cell[8] in transmission mode at BM26A Dutch-Belgian Beamline at the European Synchrotron Radiation Facility (ESRF). The cycling rate used for the *in situ* experiment was C/2, as imposed by the short time available at the beamtime. The energy was tuned via a double-crystal monochromator operating in fixed-exit mode equipped with a Si (111) crystal pair. Absorption intensity was measured with ion chambers. Energy calibration was performed by measuring XAS of iron metal foil. In total almost 40 spectra, five spectra per hour, were acquired while the FeNCN electrode was subjected to electrochemical discharge vs. Li.

The whole *operando* Extended X-ray Absorption Fine Structure (EXAFS) dataset was globally analyzed using a chemometric approach described in detail elsewhere.[9] In short, the complete series of spectra measured along the lithiation was first analyzed by Principal Component Analysis (PCA), a factor analysis tool generally used to elucidate the minimal particular structures in multivariate spectral data sets.[10] PCA is used here to determine the number of independent components contributing to the whole series of spectra collected during electrochemical cycling. The number of principal components determined in this way is subsequently used as the basis for

multivariate curve resolution-alternating least squares (MCR-ALS) analysis.[11] This algorithm allows the stepwise reconstruction of the "pure" spectral components which are necessary for interpreting the whole set of *operando* spectra. The MCR-ALS analysis was carried out with the following constraints: non-negativity of the concentration of the components, closure (sum of the components concentrations equal to 100%), as well as unimodality of all *three* components, reflecting the irreversible transformation of the pristine material. Moreover, the presence of a single component at the beginning and end of the discharge reaction was enforced.

The pure spectral components reconstructed by MCR-ALS were subsequently fitted as classical EXAFS spectra using the Demeter software package.[12] Fourier transforms of EXAFS oscillations with different k weights were carried out from 0.25 to 0.95 nm^{-1}. Fitting was performed in the R-range from 0.1 to 0.35 nm using k^1, k^2 and k^3 weights. EXAFS amplitudes and phase-shifts were calculated using the program FEFF7[13] starting from the calculated lattice parameters of FeNCN and of the intermediate composition $Li_2Fe_2(NCN)_3$. The structure of the latter was obtained by ion exchange of a Li_2NCN supercell. The amplitude reduction factor S_0^2 was fitted in the spectrum of pristine FeNCN to the value of 0.95, and used as an internal standard. In the fitting of the other spectra, S_0^2 was kept fixed to this value. In the case of spectra containing signals originating from multiple phases, the sum of the S_0^2 of each phase was made equal to 0.95. The coordination numbers N were kept fixed during the fitting, whereas interatomic distances (R) and the Debye-Waller factors (σ^2) were calculated for all paths included in the fits. These parameters are reported in Table S3.

2. Choice of PBE+*U* model

The cell voltage is one of the most important parameters for any Li/Na-ion battery. To start with, we calculated the average cell voltages of the TMC against the pure Li/Na metals within the PBE and PBE+*U* density-functional parameterizations. To examine the influence of the magnetic state and orderings on the cell voltage, both ferromagnetic and antiferromagnetic models within PBE+*U* were taken into consideration. Experimentally, the cell voltage is the arithmetic average of the two potentials evaluated at half-charge and half-discharge, as depicted in Figure S1 and numerically tabulated in Table S2. The experimental voltage profiles shown in Figure S1 were obtained at the current rate of C/10, with the exception of those of FeNCN in Li-ion and Na-ion batteries that were measured at C/50 and C/5, respectively. We defined the voltage error as the voltage difference between the calculated and experimental data because it provides useful guidance in the proper choice of the simulation parameters, as shown in Figure S2.

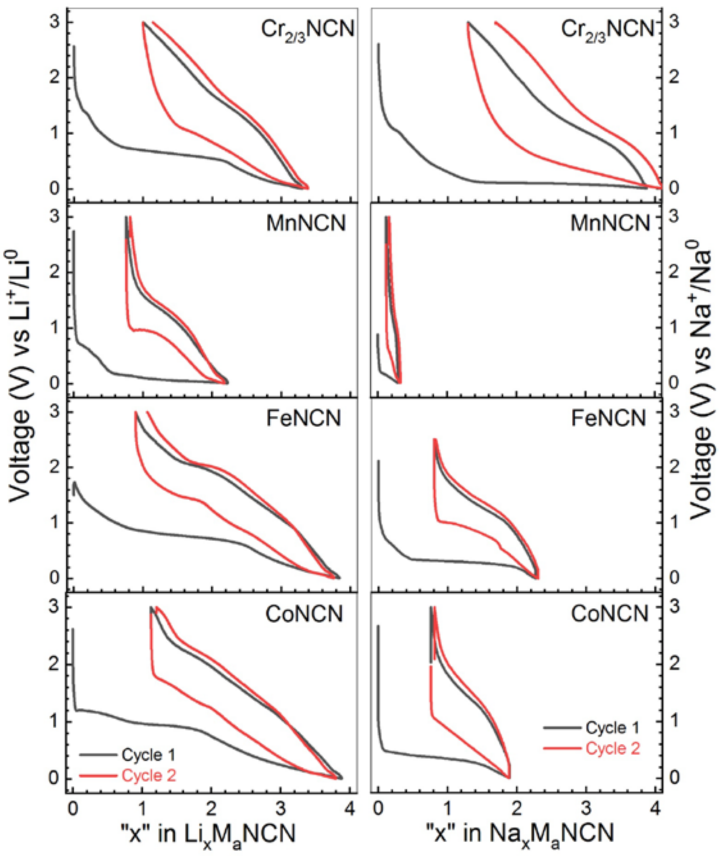

Figure S1. Experimental voltage profiles of carbodiimides in Li/Na-ion batteries.

Table S2. Experimental and theoretical cell voltage data of carbodiimides in Li/Na-ion batteries.

Batteries	Structures	Averaged cell voltage (V)			
		exp	PBE	PBE+*U*	PBE+*U* (AFM)
Li-ion	Cr_2NCN_3	1.1	1.6	0.9	0.9
	MnNCN	0.9	1.7	1.0	1.0
	FeNCN	1.4	2.2	1.6	1.6
	CoNCN	1.2	2.4	2.0	2.0
Na-ion	Cr_2NCN_3	0.9	1.1	0.4	0.4
	MnNCN	0.8	1.2	0.5	0.5
	FeNCN	1.0	1.7	1.1	1.1
	CoNCN	1.0	1.9	1.5	1.5

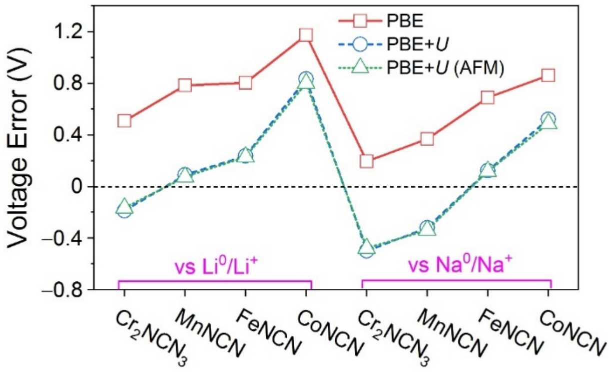

Figure S2. The relative error in the lithiation/sodiation potential within PBE and PBE+*U* in both ferromagnetic (FM) and antiferromagnetic (AFM) models, as compared with the experimental cell voltages.

Generally, pure PBE produces inferior voltage data (the largest error being 1.2 V for CoNCN in a LIB), because it doesn't suffice for transition-metal compounds in which the electrons trend to be highly localized and interacting.[14] After introducing the *U* correction, the errors decrease significantly (to about 0.8 V for CoNCN in a LIB), the only exception being $Cr_2(NCN)_3$ in a NIB but barely so. Still, there is scatter around the zero line. In addition, there is negligible voltage difference when comparing the antiferromagnetic from the ferromagnetic model, so it reasonable and economic, too, to choose the faster ferromagnetic models within PBE+*U* for a large-scale study. In addition, the voltage increases with the increasing electronegativity of the metal (from Cr to Co), probably originating from the increasing covalency of the metal–nitrogen bonds in the carbodiimides.[15]

3. Calculated theoretical phase diagram

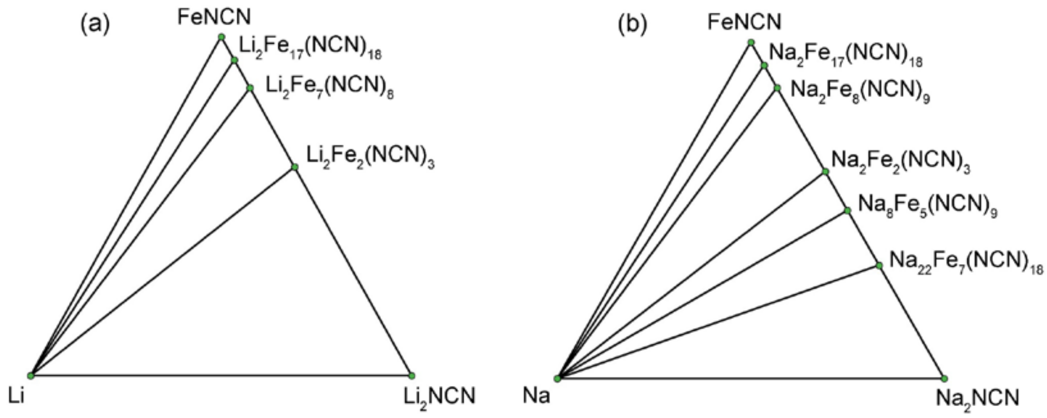

Figure S3. Phase diagrams of (a) Li–Fe–NCN (in a LIB) and (b) Na–Fe–NCN (in a NIB) ternary compounds.

4. *Operando* XAS data analysis

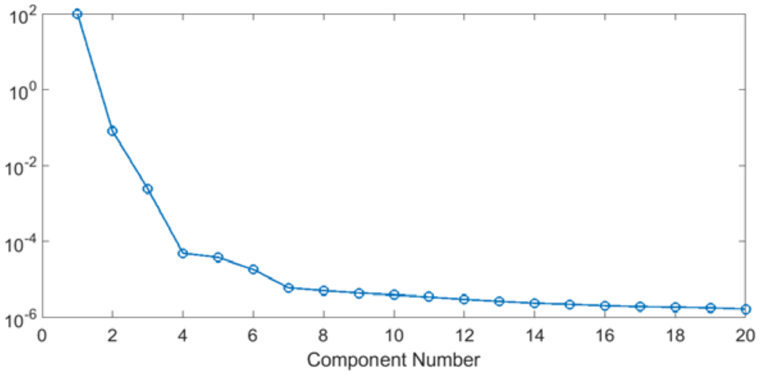

Figure S4. Variance obtained via PCA analysis applied to XAS data set reveals that 99.9% of total variance is comprised by three principal components.

After careful alignment and normalization, the presented dataset was subjected to PCA analysis, which points towards the prevalence of *three* independent principal components comprising the entire data set, see Figure S4. The information obtained from the rank analysis is particularly interesting, since it means that at least *one* intermediate component is necessary to interpret the whole set of data. This result was subsequently used along with chemical constraints (described in the experimental section) to run MCR-ALS analysis. The pure spectral components along with their evolution during electrochemical discharge are presented in Figure S5(a) and (b), respectively.

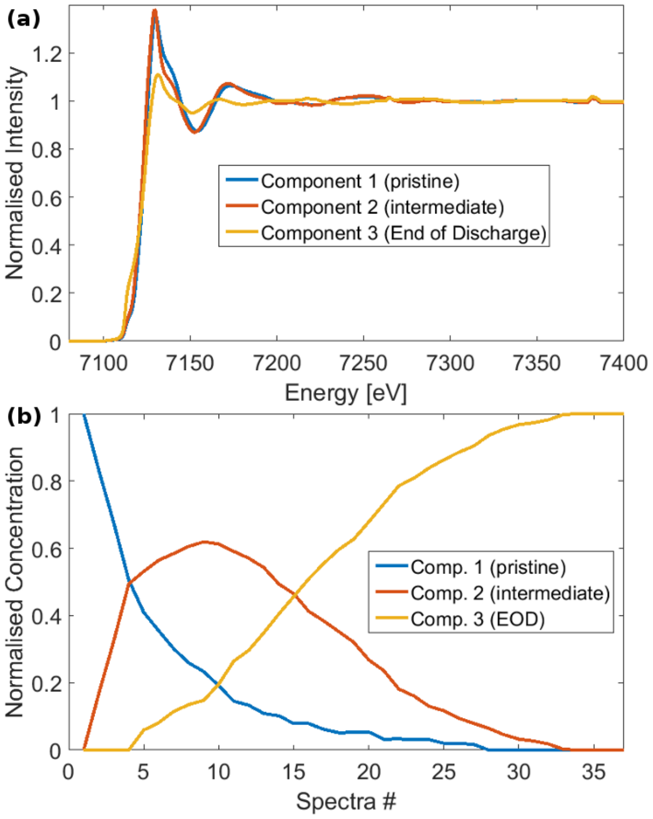

Figure S5. a) Pure spectral components obtained via MCR-ALS and b) the evolution of their concentration upon electrochemical lithiation of FeNCN.

The EXAFS spectrum of component 1, which represents 100% of the spectrum of pristine FeNCN, can be easily fitted with the structure of FeNCN (Figure S6). Component 3, representing the sample at the end of the discharge, is composed primarily of a single Fe–Fe shell with reduced coordination number compared to Fe metal, thus suggesting the prevalence of iron nanoparticles. Moreover, a slight contribution of a Fe–N shell is needed for a satisfying fit, which indicates that the reduction reaction was not completed in the *in situ* cell (Figure S7)

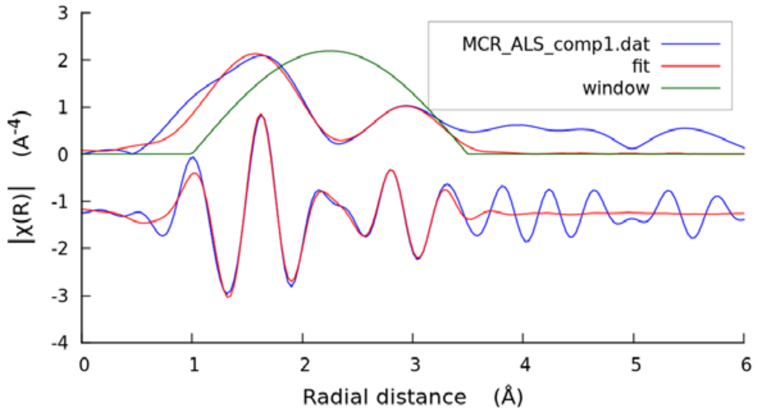

Figure S6. Fourier transform of the Fe K-edge EXAFS spectrum of pristine FeNCN (component 1) obtained via MCR-ALS and EXAFS fit with fitting window.

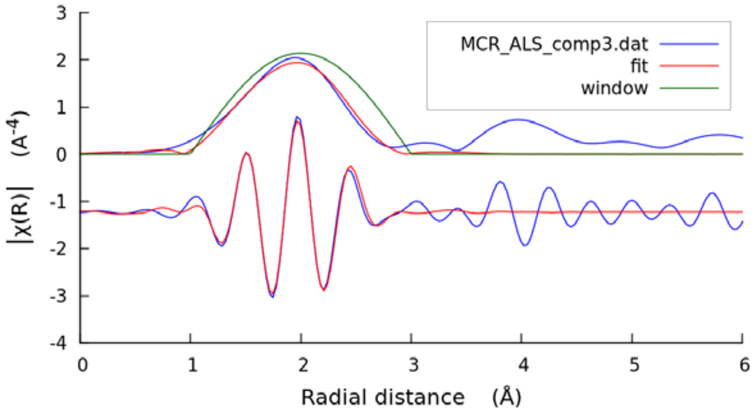

Figure S7. Fourier transform of the Fe K-edge EXAFS spectrum of EOD component 3 obtained via MCR-ALS and EXAFS fit with fitting window.

Table S3. EXAFS fitting parameters of MCR-ALS component 1 (pristine) of Fe K-Edge

Shell	Coord. no.	theoretical distance (Å)	exp. distance (Å)	σ^2
Fe–N	2.5	2.20	2.02(1)	0.004(3)
	3.5		2.198(6)	
Fe–C	6	3.01	3.00(3)	0.024(6)
Fe–Fe	6	3.27	3.29(1)	0.037(5)

Table S4. EXAFS fitting parameters of MCR-ALS component 3 (EOD) of Fe K-Edge

Shell	Coord. no.	theoretical distance (Å)	exp. distance (Å)	σ^2	amp
Fe–N	6	2.20	2.06(1)	0.009(2)	0.21
Fe–Fe	6	2.482	2.468(8)	0.024(3)	0.74

5. Chemical bonding analysis of $Li_2Fe_2(NCN)_3$ models

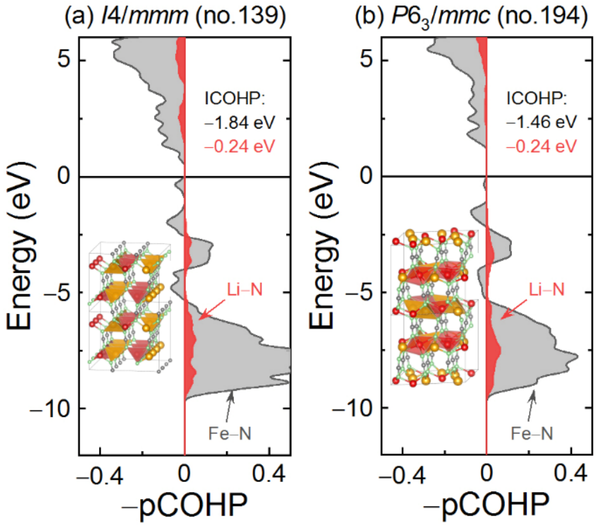

Figure S8. Chemical-bonding analysis of two $Li_2Fe_2(NCN)_3$ structural models derived from (a) Li_2NCN (*I*4/*mmm*) and (b) FeNCN (*P*6_3/*mmc*) supercells. The Fermi level is set to zero. The ICOHP of Fe–N in (a) is much more negative than that in (b), indicating that (a) is the preferred structure (stronger bonding) for the $Li_2Fe_2(NCN)_3$ composition.

6. References

[1] G. Kresse, J. Furthmüller, *Phys. Rev. B.* **1996**, *54*, 11169-11186.
[2] A. Jain, G. Hautier, S. P. Ong, C. J. Moore, C. C. Fischer, K. A. Persson, G. Ceder, *Phys. Rev. B.* **2011**, *84*, 045115.
[3] S. P. Ong, W. D. Richards, A. Jain, G. Hautier, M. Kocher, S. Cholia, D. Gunter, V. L. Chevrier, K. A. Persson, G. Ceder, *Comp. Mater. Sci.* **2013**, *68*, 314-319.
[4] A. Urban, D.-H. Seo, G. Ceder, *npj. Comp. Mater.* **2016**, *2*, 1-13.
[5] R. P. Stoffel, C. Wessel, M. W. Lumey, R. Dronskowski, *Angew. Chem. Int. Ed.* **2010**, *49*, 5242-5266.
[6] a) S. Maintz, V. L. Deringer, A. L. Tchougréeff, R. Dronskowski, *J. Comput. Chem.* **2016**, *37*, 1030-1035; b) W. L. Li, C. Ertural, D. Bogdanovski, J. Li, R. Dronskowski, *Inorg. Chem.* **2018**, *57*, 12999-13008; c) S. Maintz, V. L. Deringer, A. L. Tchougréeff, R. Dronskowski, *J. Comput. Chem.* **2013**, *34*, 2557-2567; d) R. Dronskowski, P. E. Blöchl, *J. Phys. Chem.* **1993**, *97*, 8617-8624; e) V. L. Deringer, A. L. Tchougréeff, R. Dronskowski, *J. Phys. Chem. A.* **2011**, *115*, 5461-5466.
[7] X. Liu, L. Stork, M. Speldrich, H. Lueken, R. Dronskowski, *Chem. Eur. J.* **2009**, *15*, 1558-1561.
[8] J. B. Leriche, S. Hamelet, J. Shu, M. Morcrette, C. Masquelier, G. Ouvrard, M. Zerrouki, P. Soudan, S. Belin, E. Elkaim, F. Baudelet, *J. Electrochem. Soc.* **2010**, *157*, A606-A610.
[9] M. Fehse, A. Iadecola, M. T. Sougrati, P. Conti, M. Giorgetti, L. Stievano, *Energy Storage Materials* **2019**, *18*, 328-337.
[10] D. L. Massart, B. G. M. Vandeginste, L. M. C. Buydens, S. De Jong, P. J. Lewi, J. Smeyers-Verbeke, *Handbook of Chemometrics and Qualimetrics: Part A, Vol. 20*, Elsevier B.V., **1997**.
[11] a) J. Jaumot, R. Gargallo, A. de Juan, R. Tauler, *Chemometr. Intell. Lab.* **2005**, *76*, 101-110; b) J. Jaumot, A. de Juan, R. Tauler, *Chemometr. Intell. Lab.* **2015**, *140*, 1-12.
[12] B. Ravel, M. Newville, *J. Synchrotron. Radiat.* **2005**, *12*, 537-541.
[13] A. L. Ankudinov, J. J. Rehr, *Phys. Rev. B.* **1997**, *56*, R1712-R1716.
[14] Y. S. Meng, M. E. Arroyo-de Dompablo, *Energ. Environ. Sci.* **2009**, *2*, 589-609.
[15] M. K. Aydinol, A. F. Kohan, G. Ceder, K. Cho, J. Joannopoulos, *Phys. Rev. B.* **1997**, *56*, 1354-1365.
